# Unveiling the Drug Formulation Code: A Journey to Three-Dimensional Precision

**DOI:** 10.7759/cureus.62614

**Published:** 2024-06-18

**Authors:** Esteban Zavaleta-Monestel, Monserrat Barrantes-López, Jonathan García-Montero, Sebastián Arguedas-Chacón, Jeimy Campos-Hernández

**Affiliations:** 1 Pharmacy, Hospital Clínica Bíblica, San José, CRI; 2 Pharmacy, Universidad de Iberoámerica, San José, CRI; 3 Pharmacy and Clinical Research, Hospital Clínica Bíblica, San José, CRI

**Keywords:** three-dimensional (3d) printing, technology, personalized treatment, magistral formulation, pharmaceutical form

## Abstract

Magistral formulations emerged years ago and were of great help in the personalization of treatments for patients. Over time, innovation began in this area with new technologies such as three-dimensional (3D) printing, which has brought greater benefits, ease of preparation, new scopes, and even cost reduction. Three-dimensional printing of medicines opened the way to create personalized multi-dose, controlled-release, multi-drug tablets, among others. In addition, this technology manages to be more specific in adjusting pharmacokinetics, doses, and even organoleptic qualities, which is precisely what is sought since the medication is being personalized for a patient due to a particular case or condition. Throughout the research, some studies can be observed that function as a base that provides safety and effectiveness for the subsequent use of other pharmaceuticals in the 3D printing of medicines.

## Introduction and background

In the field of science, new advances, discoveries, and technologies are emerging every day. Three-dimensional (3D) imaging is a clear example of this and has come to represent not only a great advance but also greater ease of use and new applications throughout the scientific field. This technology has evolved in 3D printing, achieving the production of organs, tissues, drugs, and prostheses, among many other medical applications, being of great help to the medical and pharmaceutical sectors mainly [1].

Three-dimensional printing can be defined as the set of technologies that manage to create different solid and 3D objects of any shape from digital models. These results of physical objects are obtained from a procedure that has several steps in which a superposition of layers of certain materials ends up being produced, and for this, it is also necessary to have different software and hardware [2,3]. When referring to the steps or stages of 3D printing, it can be defined that, first, the model must be designed digitally with the help of computer-aided design software. Next, this model is passed through a slicing program that manages to divide the figure into layers and then instructs the printer on how the physical object is required to be created. Finally, after configuring the printer, it adds layer by layer to the model for a final product that will then be dried, sanded, and refined [4].

Three-dimensional printing has found numerous applications in the healthcare field, particularly in the development of personalized treatments for patients. For instance, it can be used to customize pre-surgical procedures, reducing operating and recovery times. Additionally, it can be used to create personalized synthetic organs, shorten transplant waiting lists, and fabricate custom prostheses [5]. Three-dimensional printing has also made significant contributions to the pharmaceutical sector. This technology has enabled the development of medications with specific pharmacokinetics and properties tailored to individual patients or treatments. In 2015, the FDA approved the first 3D-printed drug, Spritam, for the treatment of epilepsy. This drug was a buccally dispersible formulation of levetiracetam and marked the beginning of the development of various controlled-release dosage forms, multi-dose systems, and others [6].

It is important to note that the preparation of a medication, also known as a magistral formulation, is essential as it always seeks to adapt to the pharmacological and clinical needs of a patient [7]. Thanks to this area, personalized pharmaceutical preparations are made and now face new possibilities and facilities due to 3D printing and its application in the pharmaceutical sector [8].

The combination of magistral formulations and 3D printing has had a profoundly positive impact on the healthcare sector, and its potential will continue to expand as knowledge advances. This technology has not only enabled personalized treatments but has also demonstrated the potential to reduce costs, increase accessibility, and enhance personalization [9]. This study aims to elucidate the mechanisms and impact of 3D printing in the pharmaceutical sector.

## Review

In addition to the 3D printing process, it is important to note that there are different printing techniques depending on the desired outcome. These techniques are differentiated based on three aspects: the materials required, the method of layer creation, and the method of layer bonding [10]. Table 1 summarizes the seven types of 3D printing that can be used [10-13].

**Table 1:** Types of 3D printing techniques LENS: laser engineered net shaping, EBAM: electron beam additive manufacturing, FDM: fused deposition modeling, PAM: pressure-assisted microsyringe, MJ: material jetting, NPJ: nanoparticles jetting, DoD: drop on demand, BJ: binder jetting, SLA: stereolithography, DLP: digital light processing, CDLP: continuous digital light projection, MJF: multi-jet fusion, SLS: selective laser sintering, SLM: selective laser melting, EBM: electron beam melting, LOM: laminated object manufacturing

Types of impressions	Characteristics
Direct energy deposition	Uses different types of metals as a base (titanium, stainless steel) and uses a laser to make the parts. It is divided into the LENS and EBAM types.
Material extrusion	Works with thermoplastic materials and is known as fused filament fabrication. It is divided into the FDM and PAM types.
Material injection	It is capable of printing designs in multiple colors or materials in a single print. It works with materials that are photopolymers or stainless steel and has the types MJ, NPJ, and DoD.
Binder jetting	It works with materials such as plaster and ceramics and only has the BJ type. It is an additive manufacturing process.
Photopolymerization	It has several processes one based on photopolymer resins that are thermoset. It is divided into SLA, DLP, and CDLP.
Powder bed fusion	It is an additive technology that uses heat to fuse particles of atomized powder. It works with polyamide, thermoplastics, and metal alloys. It is divided into MJF and SLS and SLM and EBM.
Sheet lamination	It can work on paper, metal, and polymers. It only has one type which is the LOM.

As can be observed, each technique has distinct characteristics. However, one of the most widely used techniques for 3D printing is material extrusion. This type of 3D printing has already been successfully employed in the creation of organs or tissues such as heart valves, skin, livers, and others. While all techniques are functional, a crucial factor in selecting the appropriate technique is not only the ability to create the organ or tissue but also the ability to print human cells to ensure safer acceptance by the body [14].

The material extrusion printing technique encompasses two subcategories: fused deposition modeling (FDM) and pressure-assisted microsyringe (PAM). Both types offer significant advantages that make them suitable for drug manufacturing. The following table summarizes the specific advantages of these two techniques [10].

**Table 2:** Main 3D printing techniques used in drug elaboration FDM: fused deposition modeling, PAM: pressure-assisted microsyringe, 3D: three-dimensional

Main 3D printing techniques	Advantages
FDM	More affordable equipment
	No post-printing procedures required
	Capable of printing multiple polymers and using solvents
	Uniformly incorporates the active ingredient
PAM	Capable of printing at room temperature, making it useful for thermolabile active ingredients
	Biocompatible materials can be used
	Higher active ingredient loads are achieved

**Figure 1:**
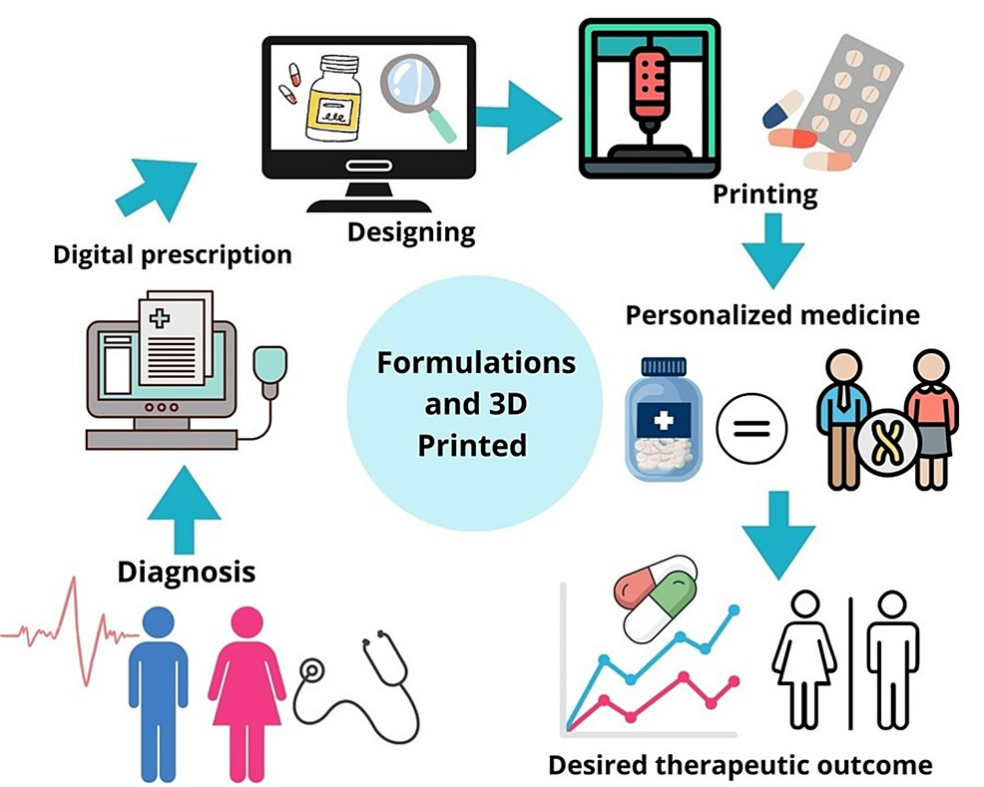
Flowchart illustrating the formulations and 3D-printed drug process Image Source: Lamichhane et al., 2019 [15]; open access

Figure 1 below shows a flowchart illustrating the origination process for the formulation and printing of a pharmaceutical drug.

As depicted in Figure 1, the process commences with a diagnosis by a medical professional. This diagnosis sparked the concept of personalized therapy, which subsequently materialized through digital prescriptions. Leveraging 3D technology, the requisite pharmaceutical form can be designed, tailoring the therapy to each patient's specific needs. Three-dimensional-printed medications ensure optimal therapeutic efficacy. This personalization mitigates the risk of overdose, enhancing patient safety and treatment outcomes, which must be meticulously evaluated to achieve the desired therapeutic effect. Notably, patients with multiple comorbidities often require multiple medications, typically necessitating dose adjustments during follow-up visits based on clinical progress. This presents a challenge in the application of 3D printing for various drug formulations [15,16].

Applications in the pharmaceutical sector

As mentioned earlier, various healthcare sectors have benefited from this technology; however, the pharmaceutical sector is of particular interest in this study. The terms "3D printing" and "medications" have appeared in scientific research since the 1980s, emphasizing the potential of this technology. Today, it is a reality that allows pharmacists to modify the design or manufacture of medications digitally [17].

Over time, the use of 3D-printed medications has shown favorable results, enabling continuous innovations in the creation of pharmaceutical forms. Currently, controlled-release systems, multi-dose systems, multi-drug tablets, and personalized pharmaceutical forms have been developed, having a positive impact on patient health and treatment [18].

In controlled-release medications, the goal is to release the active ingredient into the body at different times, prolonging its effectiveness. By using 3D printing to create these medications, an optimal shape and structure can be designed, resulting in a capsule composed of three elements: a surface erosion matrix containing the active ingredient, another without the drug, and a biodegradable layer [19]. By manufacturing a tablet in this way, it is achieved that it degrades and dissolves gradually in the body. This technology allows controlling the kinetics and, specifically, the release of the drug through the surface erosion matrix, where the active ingredient is found. [20] Thanks to 3D printing of controlled-release drugs, a significant increase in bioavailability and prolonged release have been achieved compared to existing commercial preparations [6].

On the other hand, there are multi-dose systems, which are those that have different drug doses and different releases. One case where the effectiveness of such a drug can be verified by means of 3D printing is with glipizide. The aim was to obtain filaments loaded with different concentrations of the drug and thus achieve two types of distribution. This is how Duo Tablets emerged, where this technology achieved a diffusion release for the active ingredient and dissolution of the polymer at different times [21]. Likewise, there are multi-drug tablets where, thanks to 3D printing, it was possible to create a pill that, in this case, contained three different drugs with two different mechanisms of action as well. In the case of one study, a multi-drug was created for patients with diabetes and hypertension, so the drug contained an ACE inhibitor, a sulfonylurea, and a calcium channel blocker. With the help of 3D printing, it was possible to design a multi-compartment system and, through layer separation, to obtain different release mechanisms [22]. By creating a multi-compartment tablet, interactions between active ingredients are avoided, and the patient only takes one tablet instead of three [23].

As for personalized dosage forms, although these types of treatments have existed for many years, it has been discovered that it is also possible to make them using 3D printing technology [24]. This allows adjusting the doses, pharmacokinetics, adverse effects, and therapeutic response to adapt to each patient, thus providing more effective treatment [25,26]. The profitability of 3D formulations will depend on the type of formulation desired and the type of technique and printer to be used. For instance, FDM printers have been found to be the least expensive, ranging from, for example, 500 to 2000 pounds sterling, or approximately $637 to $2548 at today's exchange rate. This, coupled with the type of formulation and the company that manufactures them, means that the total individualized costs per patient can become high, becoming a limitation for many patients. However, it is important to mention that their potential level of effectiveness also offers a good alternative for the creation of personalized pharmaceutical forms, so the cost-effectiveness evaluation should always be valued and represents a great challenge in formulation [17,27].

According to a study conducted in pediatric patients with maple syrup urine disease, a very specific dose is required that depends on weight, age, and blood levels. For this reason, personalized isoleucine treatments were tested using 3D printing, which was simpler. This technology allowed the establishment of precise ranges of micromoles required for each dose, and the printer followed these indications to create the medication, thus facilitating the obtaining of accurate results [28].

An important aspect of the creation of pharmaceutical forms, such as multi-compartment or multi-dose systems using 3D printing technology, is the reduction of interactions between active ingredients. This is because each pharmaceutical preparation is made with a specific system to minimize these interactions. In polymedicated patients, many interactions occur at the pharmacokinetic level, affecting the therapeutic effect of some drugs [29]. With 3D printing, active ingredients can be placed in different layers of the tablet and released at different times, thus reducing possible interactions.

Structure and properties of drugs

Each drug created has its own pharmacokinetic, pharmacodynamic, oligomeric, and other properties. In order to prepare a magistral formulation for a specific patient, these properties must be altered, so it is essential to be able to know more about the structure and properties of drugs before making any adjustments to them, as is done in personalized 3D printing treatments for drugs.

At the beginning of drug creation, the potency of the drug was more important than the properties of the drug itself. However, over time, it was understood that both qualities are important for a safe and effective outcome. Taking into account aspects such as solubility, permeability, ADME (absorption, distribution, metabolism, and elimination), bioavailability, and toxicity are essential when developing a drug for marketing. If these aspects are not taken into consideration, the drug may have a shorter half-life or bioavailability, with a higher rate of interactions, and all of this may affect the desired result [30].

Controlled drug delivery systems have revolutionized pharmacology, overcoming the limitations of conventional methods. Thanks to innovative technologies such as 3D printing, drug release becomes a precise and personalized process, adapting to the specific needs of each patient. Three-dimensional printing allows the manufacture of release systems with complex geometries, meticulously controlling the speed and location of drug release [31]. By personalizing doses, not only patient health but also adherence are benefited. By receiving a treatment designed specifically for their needs, the patient is more likely to follow medical instructions correctly and consistently [31].

The possibilities of controlled drug release are further expanded with the incorporation of materials of different natures, such as lipids and polymers, in the manufacture of release systems. The creation of "layers," "multilayers," and "reservoirs" with these materials allows even more precise control of the drug release rate in the biological environment. This combination of geometry and materials allows many possibilities for the development of release systems with additional functionalities, such as flotation that allows the gastroprotection of the drug, prolonging its therapeutic window, and maximizing its effectiveness. The combinations of geometries and materials are endless, which opens up a world of possibilities for the development of new personalized, more effective, and safer treatments [32].

Advantages of 3D printing in drug formulation

**Figure 2:**
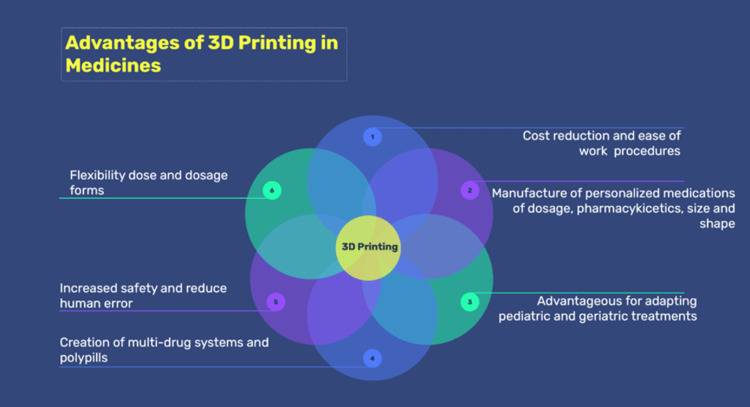
Advantages of 3D printing in medicines Image Credit: Author

Studies and the use of 3D printing technology have shown that the manufacture of drugs using this technique offers a number of advantages for both patients professionals and medical centers (Figure 2). The following table presents a list of benefits that 3D printing has brought to the pharmaceutical sector, driving innovation and the advancement of magistral formulations [33].

In the case of treatments for pediatric patients, 3D printing is extremely useful, as medications are often designed for adults and often need to be adapted for children. With 3D printing, it is possible to create pharmaceutical forms, doses, and organoleptic properties that are suitable for these patients from the outset. In addition, the color and shape of the medication can be adjusted to make it more attractive and easier for children to administer [34]. Another outstanding use of 3D printing is the manufacture of gummy pharmaceutical forms. This presentation is ideal for children, as they have a pleasant taste and, being chewable, are easier to ingest. For this type of pharmaceutical form, a semi-solid extrusion printer (PAM) is required due to the necessary viscosity. Only with this type of printer can the desired consistency be achieved in a 24-hour period [35].

Similarly, another population that greatly benefits from 3D-printed medications is the elderly. Generally, this population is often polymedicated due to the multiple pathologies they have. Therefore, many times, interactions or decreased adherence can occur due to the number of medications they must take. Therefore, being able to produce multi-dose medications or multi-drug tablets is of great benefit for this type of patient [36].

Finally, it is important to note that, along with the advantages of 3D printing, complications and doubts have also arisen along the way. As this technology has advanced, questions have arisen about the safety of the medication, its half-life, and even the temperature at which these drugs should be administered. In addition, there were initial concerns about the cost of these medications, given the use of more advanced technology. However, 3D printing has proven to be a versatile, lower-cost, and highly flexible technique, counteracting these initial concerns [37].

## Conclusions

As has been evidenced, the use of 3D printing for the manufacture of medicines in the pharmaceutical sector has brought great opportunities, innovations, and advantages. This technology allows for personalized treatments for patients, as it is possible to adjust a precise dose, change the shapes or sizes of medications, and even include more than one active ingredient in a single pharmaceutical form. Therefore, it is becoming very useful for polymedicated patients, pediatric and geriatric populations, or for specific cases of patients with a specific need. Likewise, it is a technique that has also contributed to speed, ease, and reduction in human errors and which could reduce costs in a long time, however, if it becomes a challenge for some patients to access. Finally, it is very possible that over the years it will be a technique increasingly used in the pharmaceutical sector due to its significant impact and applicability.
